# Genome-wide characterization of trichome birefringence-like genes provides insights into fiber yield improvement

**DOI:** 10.3389/fpls.2023.1127760

**Published:** 2023-03-15

**Authors:** Ziyin Li, Yuzhen Shi, Xianghui Xiao, Jikun Song, Pengtao Li, Juwu Gong, Haibo Zhang, Wankui Gong, Aiying Liu, Renhai Peng, Haihong Shang, Qun Ge, Junwen Li, Jingtao Pan, Quanjia Chen, Quanwei Lu, Youlu Yuan

**Affiliations:** ^1^ Engineering Research Centre of Cotton, Ministry of Education, College of Agriculture, Xinjiang Agricultural University, Urumqi, China; ^2^ School of Biotechnology and Food Engineering, Anyang Institute of Technology, Anyang, China; ^3^ State Key Laboratory of Cotton Biology, Institute of Cotton Research, Chinese Academy of Agricultural Sciences, Anyang, China

**Keywords:** cotton, *TBL*, expression pattern, lint percentage, WGCNA, *GH_D02G1759*

## Abstract

Cotton is an important fiber crop. The cotton fiber is an extremely long trichome that develops from the epidermis of an ovule. The trichome is a general and multi-function plant organ, and *trichome birefringence-like* (*TBL*) genes are related to trichome development. At the genome-wide scale, we identified *TBLs* in four cotton species, comprising two cultivated tetraploids (*Gossypium hirsutum* and *G. barbadense*) and two ancestral diploids (*G. arboreum* and *G. raimondii*). Phylogenetic analysis showed that the *TBL* genes clustered into six groups. We focused on *GH_D02G1759* in group IV because it was located in a lint percentage-related quantitative trait locus. In addition, we used transcriptome profiling to characterize the role of *TBLs* in group IV in fiber development. The overexpression of *GH_D02G1759* in *Arabidopsis thaliana* resulted in more trichomes on the stems, thereby confirming its function in fiber development. Moreover, the potential interaction network was constructed based on the co-expression network, and it was found that *GH_D02G1759* may interact with several genes to regulate fiber development. These findings expand our knowledge of *TBL* family members and provide new insights for cotton molecular breeding.

## Introduction

Cotton is a primary source of natural textiles (Qin et al., 2015), and cotton fiber plays an important role in modern economic activities (Zhang et al., 2008). The trichomes on the cotton seed coat, generally referred to as fibers, are the main harvest product of cotton and a valuable resource for the textile industry (Qin and Zhu, 2011). Fiber has four continuous but overlapping developmental stages: initiation, elongation, secondary cell wall deposition, and maturation (Haigler et al., 2012; Wang et al., 2019). Fiber yield is mainly determined by the number of polarly-developed epidermal cells on the ovule during fiber initiation, with each elongated cell becoming a single fiber in the future. For mature cotton bolls, the percentage of fiber is calculated as the lint percentage (LP). As an important trait of fiber yield, LP has largely been the primary focus of many studies (Su et al., 2016; Wu et al., 2016; Song et al., 2019).

With the development of cotton genomics, many genes influencing fiber yield have been identified. The collection of fiber-yield-related genes has revealed a complex regulatory landscape involving genes from various pathways (Fang et al., 2017; Du et al., 2018; Ma et al., 2018; Huang et al., 2020; He et al., 2021; Wang et al., 2022). Given that the trichome in *Arabidopsis thaliana* has a similar development pattern to fiber in cotton, genes stimulating trichome development in *A. thaliana* are considered crucial factors in cotton fiber initiation (Wang et al., 2019). However, as genes contributing to trichome development, the role of *trichome birefringence-like* (*TBL*) genes in fiber initiation has rarely been studied.

TBL is a gene family that functions in trichome development. In *A. thaliana*, *TBL3* is transcriptionally coordinated with *CESA* genes, and the knockout of *TBL3* reduces crystalline secondary wall cellulose in both the trichome and stem (Bischoff et al., 2010). The loss of *TBL34* and *TBL35* will limit the number of xylem vessels, resulting in extremely slow plant growth (Yuan et al., 2016a). In addition, there have been some studies of *tbl* mutants in *A. thaliana*, which have revealed that the deletion of the *tbl* gene leads to plant dwarfism, weak stems, and stunted growth (Bischoff et al., 2010; Xiong et al., 2013; Schultink et al., 2015). In cotton, *TBL34* was reported to improve verticillium wilt resistance (Zhao et al., 2021b). Zhang (2020) found that *GhTBL38* affects cell wall acetylation. Improving our understanding of the role of *TBL* genes in cotton fiber initiation could provide an important gene resource for molecular breeding.

In this study, we identified several *TBL* genes in four cotton species, namely *G. arboretum*, *G. raimondii*, *G. hirsutum*, and *G. barbadense*. After comprehensive characterization of the *TBLs* and quantitative trait locus (QTL) mapping interval (QTL2) of LP by our research group (Zhao et al., 2021a), we identified *GH_D02G1759* (*GhTBL82*) as a candidate gene contributing to LP. Its role has been validated by overexpression in *A. thaliana*. This genome-wide investigation of *TBLs* will provide a theoretical basis for cotton molecular breeding.

## Material and methods

### Identification of *TBL* genes and phylogenetic analysis in *Gossypium*

The protein sequences of AtTBLs were obtained from http://www.arabidopsis.org. Based on these protein sequences from the *Arabidopsis* genome, we identified TBL members among the protein sequences from four *Gossypium* genomes, namely, *G. arboreum* (A_2_), *G. raimondii* (D_5_), *G. hirsutum* (AD_1_), and *G. barbadense* (AD_2_), by sequence alignment with an e-value of 1e^−10^ (Paterson et al., 2012; Du et al., 2018; Hu et al., 2019). The protein sequences of the *TBLs* selected by alignment were submitted to the pfam database (http://pfam.xfam.org/) and SMART database (http://smart.embl-heidelberg.de/) for further confirmation (Finn et al., 2014; Letunic et al., 2015).

The *TBL* members in the four *Gossypium* species were aligned to each other by multiple sequence alignment using clusterW (http://www.ebi.ac.uk/Tools/msa/clustalw2) (Larkin et al., 2007). Based on the multiple sequence alignments, phylogenetic trees of the *TBL* members were constructed in MEGA (v.7.0) using the neighbor-joining (NJ) method (Tamura et al., 2013). Branch support was tested based on 1000 bootstrap replicates.

### Gene structure, molecular property analysis, gene duplications, and chromosomal location of the *TBL* genes in group IV

For the gene structure characterization of the *TBLs*, the gene feature files of the four *Gossypium* members were downloaded from the CottonGen database (https://www.cottongen.org). The conserved motifs in *TBLs* were identified by MEME (http://meme-suite.org/) (Bailey et al., 2009). For the protein domain analysis, the NCBI database was used to detect the protein domains in the TBLs. TBtools (v.1.100) software was used for visualization (Chen et al., 2020). For molecular property characterization, the ProtParam tool (https://web.expasy.org/) was used (Gasteiger et al., 2003).

The locations of the *TBLs* in *G. raimondii*, *G. arboreum*, *G. barbadense*, and *G. hirsutum* were displayed on the corresponding chromosomes by MapChart (v.2.2) (Voorrips, 2002). Gene duplication and syntenic regions were identified by MCScanX, and the results were visualized by TBtools (v.1.100) (Wang et al., 2012; Chen et al., 2020).

### Gene expression and weighted gene co-expression network analysis

The transcription landscape of the *TBLs* was characterized based on previously published transcriptome data (SRA; accession number SRP084203) (Lu et al., 2017). This transcriptome dataset consisted of 12 samples, including two materials (CCRI45 and MBI7747) from six different periods, 5 days post anthesis (DPA), 7 DPA, 10 DPA, 15 DPA, 20 DPA, and 25 DPA. Genes were selected by transcription abundance based on the condition that log_2_(FPKM+1)≥1. The transcription patterns of the candidate genes were visualized by the seaborn (v.0.9.0) package (installed through anaconda).

The selected genes by condition log_2_(FPKM+1)≥1 were retained for the WGCNA using R (v.4.2.1). The soft threshold was selected based on the R-square (≥0.85) and mean connectivity (≤500). With the selected soft threshold, the co-expression network was constructed and classified into several modules. The median transcription abundance of the genes in classified modules was linked to the phenotype by Pearson’s correlation analysis.

### Kyoto encyclopedia of genes and genomes enrichment analysis and interaction network construction

The KEGG enrichment analysis for genes in the candidate modules was performed on cottonFGD (https://cottonfgd.net/analyze/) with a *q*-value threshold of 1e^-5^ (Zhu et al., 2017). The results of the KEGG analysis were displayed by the matplotlib package in python.

For the construction of the potential interaction network, we calculated Pearson’s correlation coefficient as an interaction weight between the target gene and candidate genes. The interaction network construction result was inputted into Cytoscape (v.3.7.1) for visualization.

### Quantitative real-time polymerase chain reaction analysis

For qRT-PCR of the candidate genes, CCRI45 and MBI7747, which were planted in a field in Henan Province, Anyang City, were selected as plant materials. The fibers of two plant materials at 5 DPA, 7 DPA, 10 DPA, 15 DPA, 20 DPA, and 25 DPA were collected for qRT-PCR. Each sample had three biological replicates, and the samples were immediately placed into liquid nitrogen and stored in a −80°C environment. Total RNA was extracted using an RNAprep Pure Plant Kit (Tiangen, Beijing, China). The ChamQ Universal SYBR qPCR Master Mix Kit (Vazyme) was used to perform qRT-PCR with *Gh_D03G0370* (*GhActin3*) and *AT3G18780* (*AtActin2*) as internal controls.

### Isolation of the candidate gene *GH_D02G1759* and *Arabidopsis* transformation

The 1,782 bp complete coding sequence (CDS) of *GH_D02G1759* was amplified using the primers 35S::*GH_D02G1759*-F and 35S:: *GH_D02G1759*-R (**Table S1**). Then, the CDS of *GH_D02G1759* was connected to the pCAMBIA3301 vector, which was digested by NcoI and BstE II. The re-constructed vector was transferred into *Agrobacterium* (GV3101), and the recombinant plasmid GV3101 was used to infect *A. thaliana* (Col-0). After genetic transformation and selfing for three generations, T3 lines were obtained for further analysis.

### Subcellular localization analysis

To investigate the subcellular localization of the GH_D02G1759 protein, the full-length coding region of *GH_D02G1759* was inserted into the pBI121-EGFP plasmid to generate Pro35S::*GH_D02G1759*-EGFP constructs and introduced into GV3101, which were transformed into tobacco leaves. The GFP fluorescence in leaf epidermal cells was observed using a laser-scanning confocal microscope (TCS SP8, Leica, Germany).

## Results

### Genome-wide identification of *TBL* members in *Gossypium*

To identify all *TBL* members in *G. hirsutum*, *G. barbadense*, *G. arboreum*, and *G. raimondii*, we first collected the protein sequences of AtTBLs in *Arabidopsis* and aligned them to all protein sequences in the four cotton species. The protein sequences that were similar to AtTBLs were selected as candidate *TBL* members, and these were further inputted into the NCBI database for RING_Ubox confirmation. Finally, we identified 73, 73, 143, and 146 *TBL* genes in *G. arboreum*, *G. raimondii*, *G. hirsutum*, and *G. barbadense*, respectively. After the identification of *TBL* members, we evaluated the molecular properties of these *TBLs* and found that great divergence existed in aspects of protein length (from 87 to 1423 aa) and molecular mass (from 9913.21 to 160422.21 Da) (**Table S2**).

### Phylogenetic analysis of the *TBLs* in *Gossypium*

**Figure 1:**
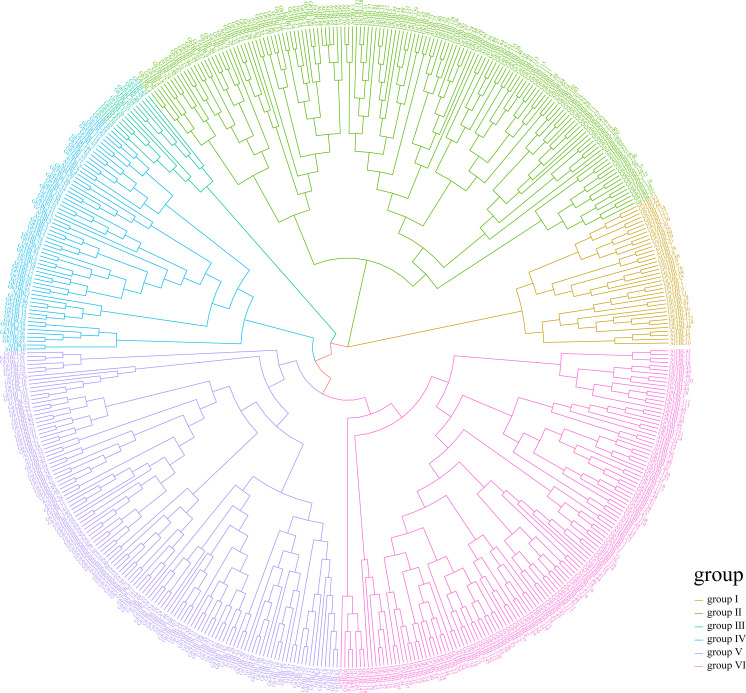
The phylogenetic analysis of TBL members across *Gossypium* and *Arabidopsis*.

Through investigating the evolutionary trajectories of *TBL* members in cotton, we built a phylogenetic tree using *TBLs* from four cotton species and *A. thaliana* (**Figure 1**). From the result of the phylogenetic tree, we noticed that the *TBL* members could be divided into six groups consistent with a previous report on *Arabidopsis* (Bischoff et al., 2010). Among the six groups, groups II, V, and VI had the most members, containing 118, 110, and 112 members, respectively. By contrast, group III had only 12 members. Based on the QTL mapping interval of LP in our research group, the candidate gene *GH_D02G1759* was identified (Zhao et al., 2021a). The homologous gene of *AT1G60790*, *GH_D02G1759*, was assigned as a member in group IV, which contained 54 members. Therefore, we focused on the *TBLs* in group IV. **Table 1** lists the specific information of the *TBL* genes in group IV from *G. hirsutum*, such as gene ID, chromosomal location, protein size (aa), and molecular weight (Da).

**Table 1:** Physico-chemical and biochemical features of the *TBL* genes in group IV from *G. hirsutum*.

Gene name	Gene ID	Chromosome location	ORF length/bp	Length/aa	MW	PI	Grand average of hydropathicity
*GhTBL3*	*GH_A01G1358*	A01:48868545-48871628 (-)	1641	546	62,597.77	9.08	−0.59
*GhTBL6*	*GH_A01G2068*	A01:112096203-112098519 (+)	1863	620	69,372.85	9.26	−0.67
*GhTBL13*	*GH_A03G1589*	A03:97111433-97113464 (-)	1107	368	41,751.49	5.63	−0.67
*GhTBL17*	*GH_A04G0655*	A04:29654483-29657155 (-)	1965	654	73,360.29	9.39	−0.65
*GhTBL39*	*GH_A08G0217*	A08:1909865-1915388 (-)	1464	487	55,779.52	9.36	−0.61
*GhTBL50*	*GH_A10G1836*	A10:96708808-96710505 (+)	1299	432	49,906.62	8.39	−0.41
*GhTBL51*	*GH_A10G1995*	A10:102361488-102363822 (+)	1659	552	63,403.42	9.20	−0.67
*GhTBL65*	*GH_A12G1412*	A12:84353958-84356229 (-)	1590	529	61,120.21	8.50	−0.67
*GhTBL72*	*GH_A13G2073*	A13:104466035-104470788 (-)	1239	412	47,839.62	8.37	−0.46
*GhTBL74*	*GH_D01G1448*	D01:29649597-29652662 (+)	1725	574	65,887.56	8.76	−0.54
*GhTBL77*	*GH_D01G2148*	D01:59449534-59451840 (-)	1851	616	68,848.35	9.22	−0.63
*GhTBL82*	*GH_D02G1759*	D02:58904942-58907596 (-)	1782	593	66,870.06	8.75	−0.62
*GhTBL93*	*GH_D04G0930*	D04:22963926-22966585 (+)	1959	652	73,186.09	9.44	−0.67
*GhTBL112*	*GH_D08G0229*	D08:1948481-1953778 (-)	1470	489	56,035.85	9.46	−0.65
*GhTBL122*	*GH_D10G1940*	D10:51445291-51446981 (+)	1299	432	49,913.65	8.21	−0.39
*GhTBL123*	*GH_D10G2095*	D10:55435864-55438247 (+)	1659	552	63,459.63	9.17	−0.62
*GhTBL136*	*GH_D12G1430*	D12:42896932-42899192 (-)	1578	525	60,665.79	8.38	−0.61
*GhTBL143*	*GH_D13G2051*	D13:58447821-58452588 (-)	1245	414	48,093.97	8.72	−0.46

“-” means reverse strand. “+” means forward strand.

**Figure 2:**
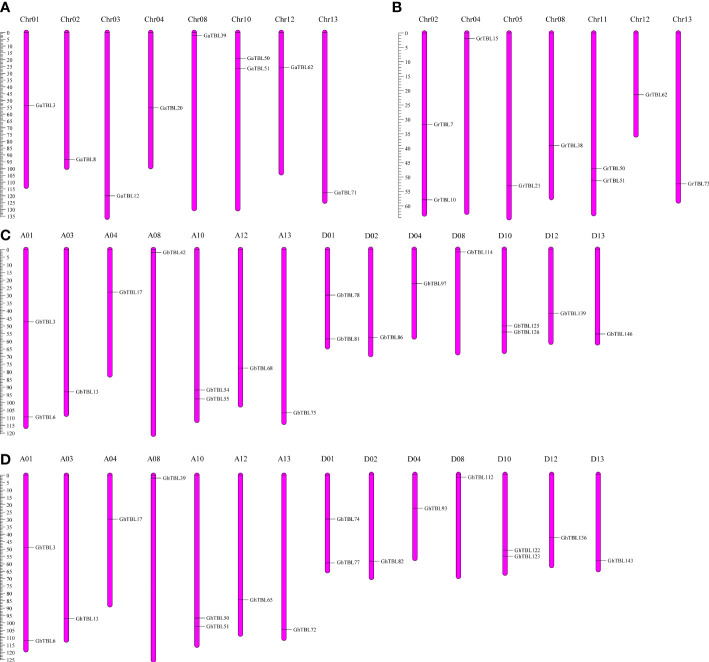
The stance of the *TBL* genes in group IV on cotton chromosomes. Chromosome distribution in *G*. *arboreum* **(A)**, *G*. *raimondii* **(B)**, *G. barbadense* **(C)**, and *G. hirsutum* **(D)**.

The group IV members were distributed in different chromosomes in the four cotton species. In *G. arboreum*, nine *GaTBLs* were located on eight chromosomes (A01, A02, A03, A04, A08, A10, A12, and A13) (**Figure 2A**). In *G. raimondii*, nine *GrTBLs* were located on seven chromosomes (D01, D02, D04, D08, D10, D12, and D13) (**Figure 2B**). In *G. hirsutum*, 18 *GhTBLs* were mapped on 14 chromosomes, including seven chromosomes from the A_t_ sub-genome and D_t_ subgenome, respectively (**Figure 2C**). The number of chromosomes containing *GbTBLs* in *G. barbadense* was the same as that in *G. hirsutum*, and the number of *GbTBLs* was also 18 (**Figure 2D**).

### Gene structure and gene duplications analysis in group IV

**Figure 3:**
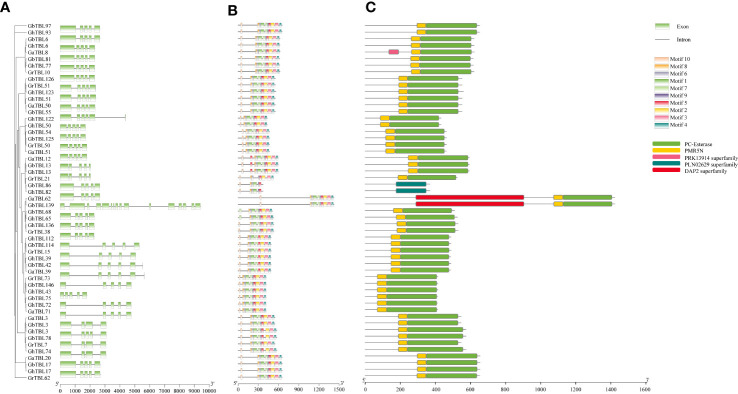
Gene structure and protein domain analysis of the *TBL* genes in group IV. The exon-intron structure **(A)** and motifs **(B)** of the *TBL* genes. **(C)** TBL protein domain prediction.

The gene structure of group IV genes showed that most *TBLs* contained five exons, a few *TBLs* contained four exons, and only *GbTBL139* had 16 exons (**Figure 3A**). Apart from gene structure, the motifs of the *TBLs* in group IV were detected by MEME, and motif detection showed that the *TBLs* had conserved motifs within gene regions (**Figure 3B**). Furthermore, we also investigated the protein domain distribution of the group IV *TBL* members (**Figure 3C**). All *TBLs* in group IV had PMR5N and PC-Esterase domains. *GbTBL139* and *GaTBL62* contained a DAP2 domain, and *GhTBL13* and *GbTBL13* contained a PLN02629 domain. From these results, we found that although most *TBL* members from group IV were conserved in cotton, a small amount of divergence remained in terms of gene structure and protein sequences.

**Figure 4:**
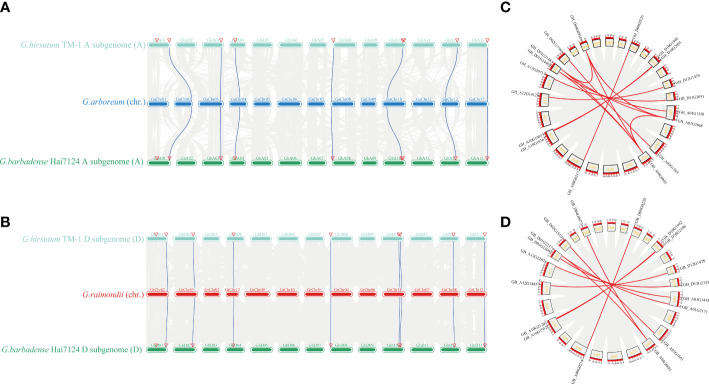
Analysis of synteny among multiple *Gossypium* genomes regarding the *TBL* genes in group IV. **(A)** Synteny analysis among *G. arboreum*, *G. hirsutum* (A_t_ subgenome), and *G. barbadense* (A_t_ subgenome). **(B)** Synteny analysis among *G. raimondii, G. hirsutum* (D_t_ subgenome), and *G. barbadense* (D_t_ subgenome). **(C)** Synteny analysis among *G. hirsutum* (A_t_ subgenome) and *G. hirsutum* (D_t_ subgenome). **(D)** Synteny analysis among *G. barbadense* (A_t_ subgenome) and *G*. *barbadense* (D_t_ subgenome).

Gene duplication events are common during plant evolution. As we found a sequence divergence in group IV members, we inferred that the *TBL* members in group IV may have experienced gene duplication events during tetraploidy. The inter-specific collinearity of the 54 *TBLs* among *G. arboreum*, *G. hirsutum*, *G. barbadense*, and *G. raimondii* was evaluated to detect gene duplication events (**Figures 4A, B**). We collected *TBLs* from *G. arboreum*, *G. hirustum* (A_t_), and *G. barbadense* (A_t_) to perform collinearity analysis on A genomes, while the same analysis was also performed on *TBLs* from *G. raimondii* and D sub-genomes of *G. hirustum* and *G. barbadense* to investigate collinearity within D genomes. We found 7 and 8 *TBLs* with collinearity within A sub-genomes and D sub-genomes, respectively (**Figures 4A, B**). The intra-specific collinearity of 54 *TBLs* among *G. hirsutum* and *G. barbadense* which includes the collinearity between A and D sub-genomes was also evaluated (**Figures 4C, D**). Both *G. hirsutum* and *G. barbadense* contained 18 *TBLs*. Most *TBLs* have good collinearity in the A_t_-genome or D_t_-genome of 2 tetraploids (**Figures 4C, D**).

### Transcription analysis for group IV *TBL* members

Although the sequence structure was thoroughly investigated, their potential roles in fiber development remained unclear. We collected published transcriptome data containing 12 samples (CCRI45 and MBI7747 from six periods) (Lu et al., 2017). CCRI45 has a high LP, while the LP of MBI7747 is relatively low. Therefore, characterizing the transcription landscape of *GhTBLs* from group IV is essential for illustrating their roles in fiber development. We filtered genes whose maximum FPKM values among 12 samples were smaller than 1, and 43,808 genes were retained. We found that some *GhTBLs* had material-specific transcription patterns, such as *GH_D01G2148* and *GH_D02G1759*, while others had stage-specific transcription patterns, such as *GH_D01G1448* and *GH_A01G2068* (**Figure S1A**). For verification of the RNA-seq results, qRT-PCR analysis was performed to quantify the differential expression of the transcripts. The overall expression levels of the six genes were consistent with the RNA-seq data, confirming that the RNA-seq data were reliable and conducive to the identification of candidate genes during fiber development (**Figure S1B**). Divergent transcription patterns of *GhTBLs* in group IV indicated that the *GhTBLs* in group IV may play multiple roles during fiber development.

**Figure 5:**
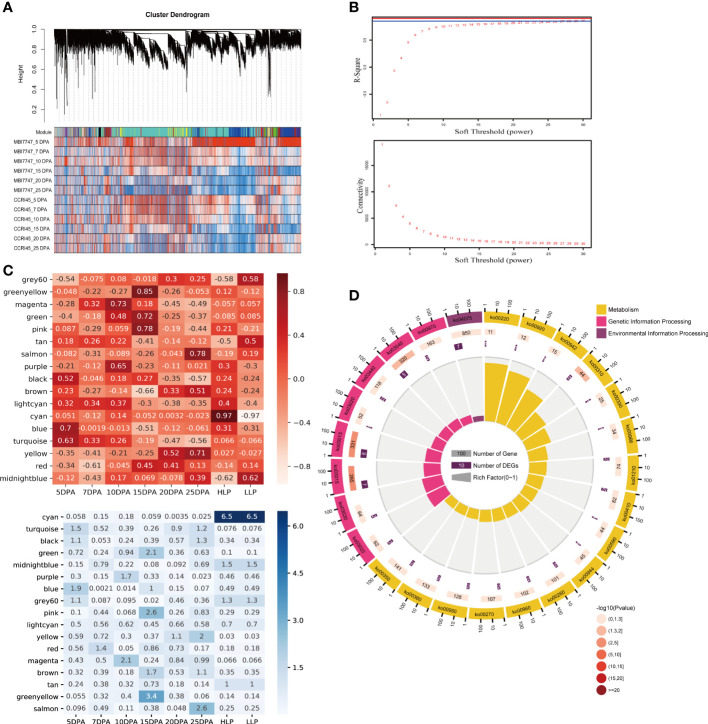
WGCNA for the transcriptome data in the study. **(A)** Results of the gene cluster analysis in WGCNA. **(B)** Mean connectivity and R-square of the WGCNA. With the increasing soft threshold, R-square rises and mean connectivity decreases. **(C)** Trait-module association results of the WGCNA. The upper heatmap is Pearson’s correlation between the modules and phenotype, and the lower heatmap is the −log_10_(*p*) value of Pearson’s significance test. **(D)** The KEGG pathway enrichment analysis of the blue module.

For further investigation of the potential association between *GhTBLs* from group IV and fiber development, we constructed a WGCNA network based on all of the retained genes (**Figure 5A**). To ensure the construction of a scale-free network, the soft threshold was selected based on both the R-square (≥ 0.85) and mean connectivity (≤ 200) (**Figure 5B**). After network construction, all of the retained genes were divided into 18 modules, which had various transcription patterns, implying complicated processes during fiber development. To dissect the associations between gene modules and fiber development, we associated the transcript abundance with the sample phenotype by Pearson’s correlation analysis. An absolute value of Pearson’s correlation coefficient larger than 0.3 and a *P*-value smaller than 0.05 were set as the threshold. We noticed that the blue and turquoise modules were linked to 5 DPA (R^2^ = 0.7 and 0.63, respectively) (**Figure 5C**). The red module was negatively related to 7 DPA (R^2^ = −0.61). The purple and magenta modules were related to 10 DPA, with R^2^ values of 0.65 and 0.73. Four modules were associated with 15 DPA, and apart from the brown module, another three modules, namely green, pink, and green-yellow, were positively related to 15 DPA (R^2^ = 0.72, R^2^ = 0.78, and R^2^ = 0.85, respectively). No modules were found to be associated with 20 DPA. Salmon and yellow were positively related to 25 DPA, while the black module was negatively associated with 25 DPA. The above modules were involved in fiber development, and other modules were found to be related to LP. The cyan module had a close association with the high LP phenotype (R^2^ = 0.97), while the midnight blue and grey60 modules were negatively correlated with high LP (**Figure 5C**).

Among the retained 14 *GhTBLs* in group IV, we found that these 14 genes were from four modules, namely the blue, turquoise, red, and yellow modules, which were involved in fiber development at different stages. Although both the blue and turquoise modules contributed to the fiber initiation stage (5 DPA), their functions differed. Genes in the blue module were mainly involved in 18 pathway categories, including 51 KEGG pathways, among which the most abundant genes were enriched in “Global and overview maps” metabolic pathways (**Figure 5D** and **Table S3**). Genes in the turquoise module had pathways related to metabolism (**Figure S2A**). Interestingly, pathways related to fatty acid biosynthesis were enriched in the turquoise modules. As fatty acids are essential for fiber elongation, we inferred that the genes in the turquoise module played an important role in fiber development. The red module was enriched in various signaling pathways, implying that this module may regulate fiber elongation by influencing multiple signaling pathways (**Figure S2B**). Regarding the yellow module, which is a module related to secondary cell wall thickening, we found that fatty acid degradation (ko00071) was significantly enriched. All of these results showed that the *GhTBLs* in the fiber-related modules had various functions in fiber development (**Figure S2C**).

### Functional validation and interaction network construction for *GH_D02G1759*

**Figure 6:**
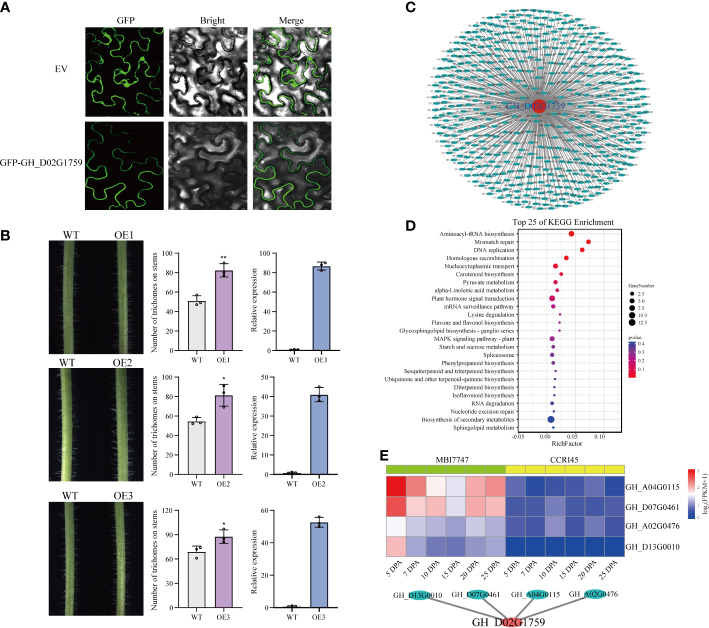
Functional investigation of the candidate gene *GH_D02G1759.* **(A)** Subcellular localization of the *GH_D02G1759* protein in the epidermal cells of the tobacco leaf. The names of the constructs are shown on the left. The scale bar is 20 μm. **(B)** Overexpression of *GH_D02G1759* in *A. thaliana.* The left images are epidermal trichomes on the stems, the middle images are the statistics on the number of trichomes (*t*-test), and the right images are the relative expression of *GH_D02G1759* in three overexpressed lines. **(C)** The entire network of *GH_D02G1759* constructed based on genome-wide transcriptome data. **(D)** The KEGG pathway enrichment analysis of the 491 genes. **(E)** The interaction network of *GH_D02G1759* constructed based on cyan module members. The heatmap indicates the transcriptional abundance of genes among samples.

*GH_D02G1759*, as the target gene, was from the blue module. The transcription patterns of *GH_D02G1759* and the blue module were similar, having high transcription abundance in fiber initiation. To address the temporal restriction of the present transcriptomic assay (http://cotton.zju.edu.cn/2.search_gene_locus.php), we checked the transcription abundance of *GH_D02G1759* from −3 DPA to 3 DPA in a previous assay (**Figure S3**). The results showed that *GH_D02G1759* was expressed in fiber initiation. We assessed the location of *GH_D02G1759 in vivo* by subcellular localization on tobacco leaves and found that *GH_D02G1759* was located on the membrane (**Figure 6A**). Furthermore, we overexpressed *GH_D02G1759* in *A. thaliana* to explore its role in trichome development. Compared with the wild type, the overexpressing plants had more trichomes on the stem surface, implying its potential role in cellular elongation. We inferred that *GH_D02G1759* could enhance the LP by stimulating the elongation of ovule epidermal cells (**Figure 6B**).

Although the function of *GH_D02G1759* has been validated by overexpression, its interaction network is still unclear. The results of the WGCNA provided us with a platform to construct a potential interaction network of *GH_D02G1759. GH_D02G1759* belongs to the blue module, and we constructed the interaction network of *GH_D02G1759* and genes from the blue modules (**Figure 6C**). A total of 491 genes from the blue module with a 0.95 Pearson’s correlation with *GH_D02G1759* were regarded as candidate-interacted genes (**Figure 6C** and **Table S4**). To detect the potential role of the interaction network of *GH_D02G1759*, we performed KEGG analysis on these 491 genes (**Figure 6D**). We found that both basic pathways (mismatch repair, DNA replication, and homologous recombination) and specific pathways (plant hormone signal transduction and starch and sucrose metabolism) were enriched in this network. Interestingly, starch is the source of fiber component synthesis, and *GH_D02G1759* may interact with genes in starch metabolism to enhance fiber development. Moreover, *GH_D02G1759* could be also regulated by genes in signal transduction pathways. There were also some secondary metabolism-related pathways in the interaction network, such as sphingolipid metabolism and flavone and flavonol biosynthesis, indicating that *GH_D02G1759* may also participate in resistance to abiotic stress (**Table S5**). As a module with a positive correlation with high LP, there were only 25 genes in the cyan module. We investigated the relationship between *GH_D02G1759* and cyan members and found that four genes, namely *GH_A02G0476* (*GhSPL7*), *GH_A04G0115* (*GhMOCS3*), *GH_D07G0461* (*GhOMA1*), and *GH_D13G0010* (*At5g05130*) were correlated to *GH_D02G1759*. *SPL7* was proved to activate miRNAs in response to several biological processes, implying that *GH_D02G1759* may influence cellular elongation *via* a complicated mechanism (**Figure 6E**).

## Discussion

As an important economic crop in the textile industry, improving the fiber yield of cotton is crucial for crop modification (Song et al., 2019). Given that fiber develops from epidermal cells, the number of epidermal cells with extreme elongation determines fiber yield (Qin et al., 2015). Therefore, stimulating the cellular elongation of epidermal cells from an ovule is a reasonable way to increase fiber yield. With the development of genomics, many large population-scale studies have been implemented to detect the functional genes involved in fiber development (Ma et al., 2018; He et al., 2021). Although a large number of functional genes have been detected, their functional validations are still limited because of the low transgenic efficiency of cotton. In response, researchers have used *A. thaliana* as a substitute to obtaining higher transgenic efficiency, as the cell structure of the trichomes in *A. thaliana* is similar to that of the fiber in cotton (Wang et al., 2004; Yang and Ye, 2013).

TBL proteins, which contribute to cellulose formation in *A. thaliana*, have been characterized (Bischoff et al., 2010). Among the 46 *TBL* members in *A. thaliana*, the functions of only a few *TBLs* have been validated. *TBL44* is related to resistance to powdery mildew fungi (Vogel et al., 2004), while *TBL3* is essential for xylan acetylation (Xiong et al., 2013; Yuan et al., 2013; Yuan et al., 2016b). In rice, *TBL1* and *TBL2* affect the acetylation level and response to rice blight disease (Gao et al., 2017). In cotton, the overexpression of *GhTBL34* can improve verticillium wilt resistance (Zhao et al., 2021b). Zhang (2020) found that the overexpression of *GhTBL38* affected cell wall acetylation. Although *TBLs* have also been found to participate in other biological processes such as pathogen response, reports on their roles during fiber development remain limited (Zhao et al., 2021a). Altogether, these studies highlight that *TBL* genes have significant value in breeding and play an important regulatory role in controlling trichome development.

We identified 73, 73, 146, and 143 *TBL* genes in *G. raimondii*, *G. arboretum*, *G. barbadense*, and *G. hirsutum*, respectively. The chromosomal distribution, evolutionary relationship, and expression patterns of the *TBL* genes in group IV were analyzed. It has been found that all *TBL* genes in group IV have typical PMR5N and PC-Esterase domains, which have acyl esterase activity and are predicted to modify cell-surface biopolymers such as glycans and glycoproteins (Anantharaman and Aravind, 2010). Gene structure analysis revealed that almost all *TBL* genes in group IV have four exons, which indicates that this gene may be functionally conserved during evolution. According to the selection pressure analysis, the Ka/Ks ratio was less than 1, further supporting the evolutionary conservation of these genes. Using published transcriptome data, we found that *TBLs* in cotton could influence fiber development *via* multiple pathways such as fatty acid biosynthesis and various signal transductions. In this study, we overexpressed the selected gene in *A. thaliana* and observed changes in the trichomes to infer the function of the candidate genes in fiber development. With the combination of transcriptome profiling and the *A. thaliana* phenotype, we confirmed the role of *GH_D02G1759* in fiber development. With the increase in the number of trichomes in the stems, we inferred that it could enhance the number of fibers on a single ovule by promoting cellular elongation. Moreover, we constructed the potential interaction network of *GH_D02G1759* based on transcription abundance. Although *GH_D02G1759* does not belong to the cyan module, which is linked to LP, it still had correlated transcription patterns with four members in the cyan module. One of these four genes, *SPL7*, activates miRNAs in response to several biological processes in which *GH_D02G1759* may participate.

All of these characterizations showed the potential role of the *TBLs* in fiber development, and the candidate gene *GH_D02G1759* detected in this study could be used as an important gene resource for improving the fiber yield of cotton.

## Data availability statement

The original contributions presented in the study are included in the article/**Supplementary Material**. Further inquiries can be directed to the corresponding authors.

## Author contributions

ZL analyzed and summarized all of the data, drew the figures, and wrote the manuscript. YS and XX participated in sample preparation. JS, PL, JG, HZ, WG, AL, RP, HS, QG, JL, and JP participated in data collection and analysis. QC, QL, and YY performed the experiments and revised the manuscript. All authors contributed to the article and approved the submitted version.
