# Supplementary figures and images for: Genome-wide characterization of trichome birefringence-like genes provides insights into fiber yield improvement
Source: Front Plant Sci. 2023 Mar 15;14:1127760. doi: 10.3389/fpls.2023.1127760 (PMC10050746; doi:10.3389/fpls.2023.1127760)

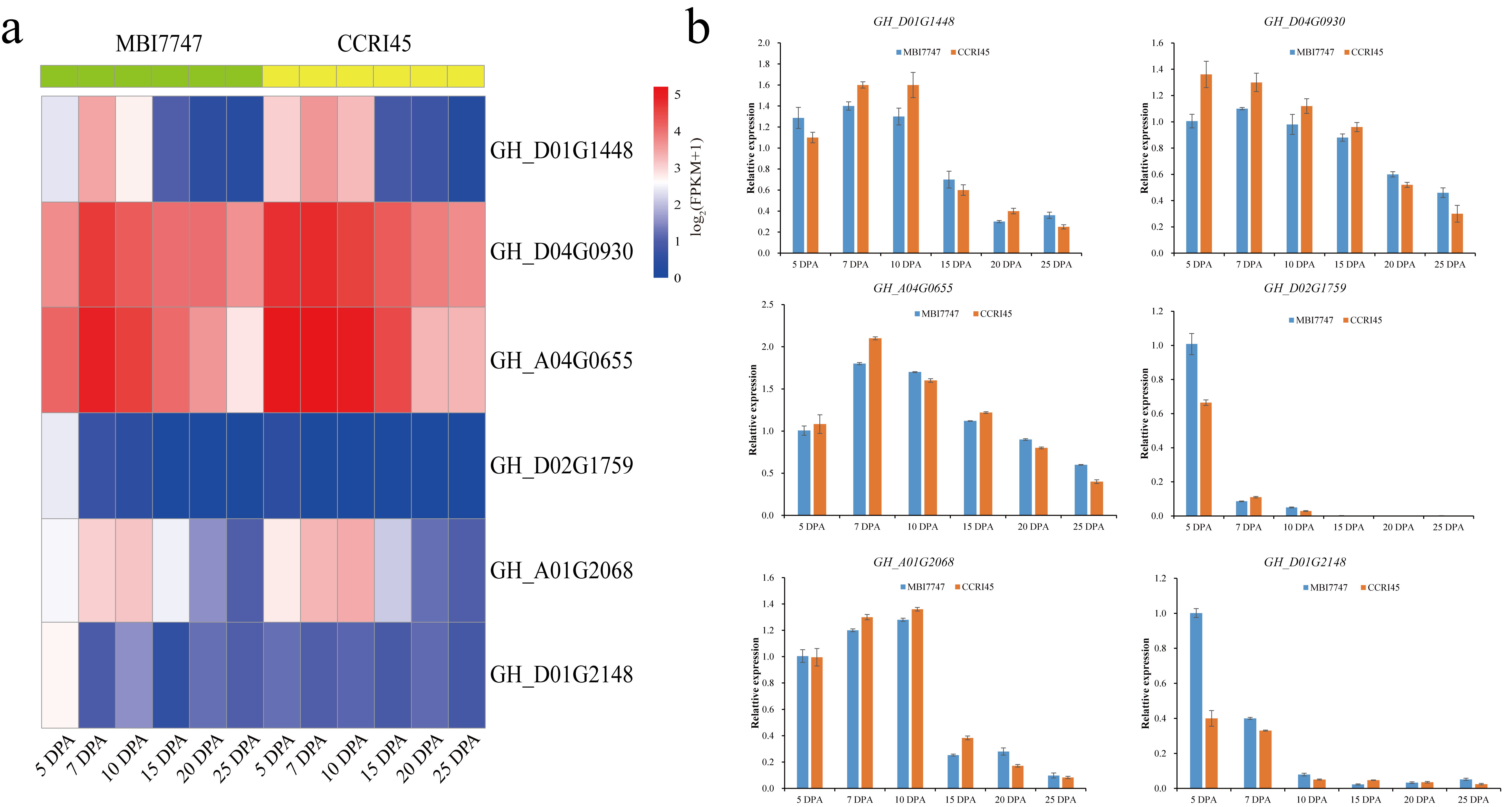

Supplement: Supplementary file 1 [file Image_1.tif]

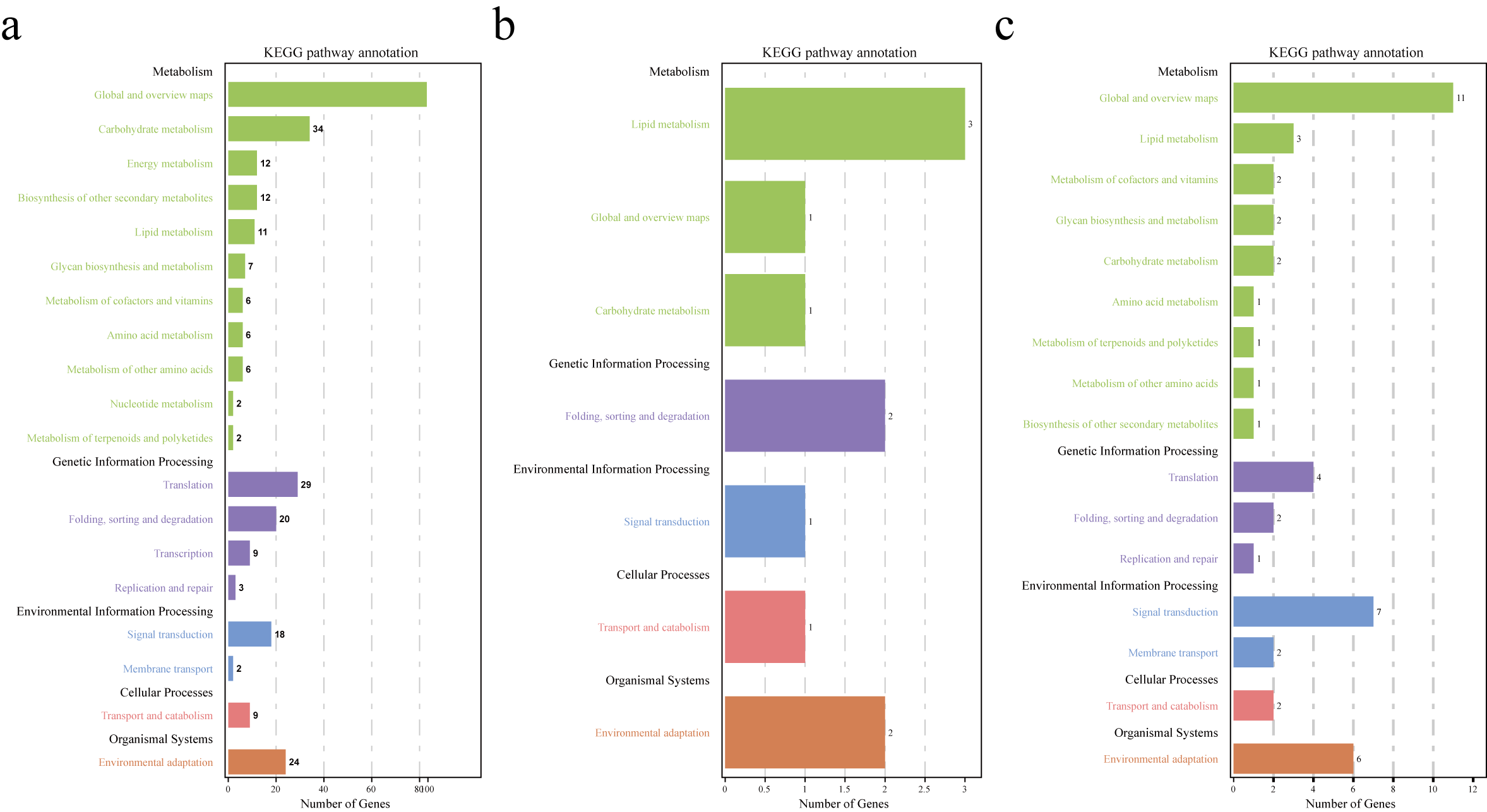

Supplement: Supplementary file 2 [file Image_2.tif]

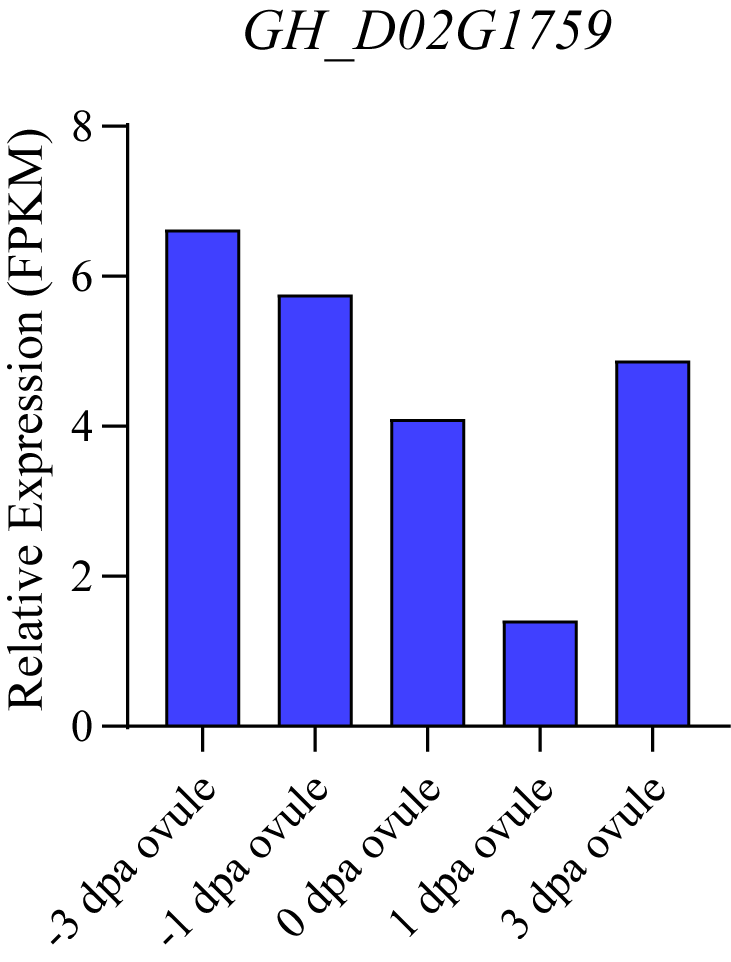

Supplement: Supplementary file 3 [file Image_3.tif]
